# Synthesis of deep-cavity fluorous calix[4]arenes as molecular recognition scaffolds

**DOI:** 10.3762/bjoc.4.36

**Published:** 2008-10-20

**Authors:** Maksim Osipov, Qianli Chu, Steven J Geib, Dennis P Curran, Stephen G Weber

**Affiliations:** 1 Department of Chemistry, University of Pittsburgh, Pittsburgh, Pennsylvania 15260, USA, Fax: +1-412-624-9861; Tel: +1-412-624-8240

**Keywords:** calixarene, fluorous, hydrofluoroethers, organofluorine, (perfluoroalkyl)alkyl aryl ethers

## Abstract

Several lower-rim perfluoroalkylated (fluorous) calix[4]arenes have been synthesized by *O*-alkylation of the parent calix[4]arene. The compounds are formed in the cone conformation. They are soluble in several fluorous solvents and show promise for use in sensing, selective extractions and other applications.

## Introduction

Calixarenes [1] are one of the most useful types of macrocyclic scaffolds. Since first reported by Zinke and Ziegler [2], calix[4]arenes have been used for a variety of molecular recognition, nanotechnology, and supramolecular applications. These have included nanowires [3], self organized nanostructures [4] chiral supramolecular assemblies [5], as well as sensors for cations [6–7], anions [8] and neutral organic molecules [9]. The versatility of the calixarene scaffold is a result of its preorganized cavity [10], which consists of four phenolic units connected by methylene bridges. Synthetic advances over the last several decades [1] have produced methodology to append various functional groups to the aromatic rings. These groups are selected to interact with specific guest molecules [11].

**Figure 1:**
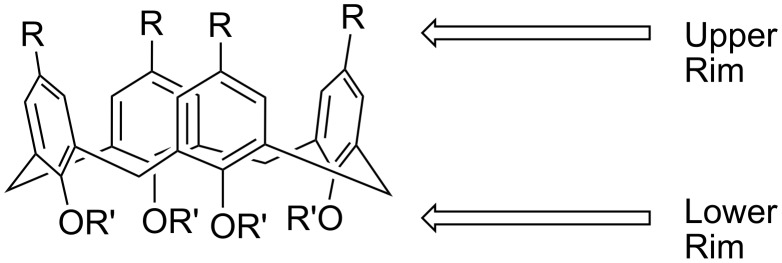
Calix[4]arene in cone conformation.

Calix[4]arenes can exist in four possible conformations: cone (Figure 1), partial cone, 1,2, and 1,3 alternates [1]. Although small groups (Me, Et) on the lower rim allow for interconversion between conformers, large groups prevent interconversion [12]. Reactions that lock the conformation result in a mixture of conformers; however, methods exist to enhance the formation of a single conformer [12]. Of the four possible conformations, the cone is the most desirable for molecular recognition and sensing applications because it has the largest available surface area for host-guest interactions [10]. With appropriate functionality and conformation, the calixarene can be tailored to bind preferentially with specific target guest molecules.

Fluorous chemistry [13] has become an increasingly popular field as a result of the multitude of applications that it has provided across the disciplines of chemistry. Fluorocarbons are extraordinarily non-polar and are at once both hydrophobic and lipophobic [14–16]. Fluorous liquids preferentially dissolve fluorous compounds and represent a unique class of selective solvents. These solvents have recently engendered powerful methods for separations [17] that have been used in applications ranging from recyclable reagents [18] to the total synthesis of natural products [19]. Fluorous compounds are the basis for highly selective ion sensors that show promise by virtue of their low level of biofouling [20]. Recently, it was shown that simple fluorous compounds act as molecular receptors for selective extraction of organic substrates into a fluorous liquid phase via hydrogen bonding [21].

Combining the selective nature of fluorous chemistry with the extensive molecular recognition capabilities of calixarenes should generate a scaffold for selective molecular receptors, yet few reports exist that detail the synthesis and applications of fluorous calixarenes [22–26]. There are no reports of studies of solubilities of such calixarenes in fluorous solvents. The work reported herein is focused on synthesizing fluorous calixarenes that are easily functionalized for selective molecular recognition and extraction of various analytes.

## Results and Discussion

**Scheme 1:**
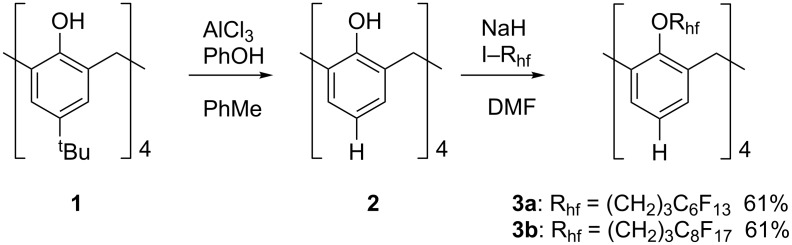
Preparation of **3a** and **3b**.

The initial target was calixarene tetra-ether **3a** bearing four perfluorohexyl groups insulated by propylene spacers. To begin, the *tert*-butyl groups were removed from commercially available 4-*tert*-butylcalix[4]arene **1**, providing calix[4]arene [27] **2**. Using NaH/DMF, conditions known to favor reaction in the cone conformation [12], **2** was alkylated with 3-(perfluorohexyl)propyl iodide to give cone conformer **3a** as the dominant tetraalkylated product in 61% yield after recrystallization (Scheme 1). However, **3a** did not exhibit the desired solubility properties and did not dissolve in perfluorinated solvents (Table 1). Therefore, to increase the fluorine content of the calixarene scaffold, **2** was treated with 3-(perfluorooctyl)propyl iodide to provide **3b** as the dominant tetraalkylated product, which was isolated in the cone conformation in 61% yield after recrystallization. Unlike the *tetra*-perfluorohexyl product **3a**, we were not able to get exact mass data for **3b** or other *tetra*-perfluorooctyl products. These compounds are otherwise well characterized and structures and purities are secure (see Supporting Information File 1).

The solubility of **3b** was explored in a variety of organic and fluorous solvents (Table 1). As with many calixarenes, **3b** was highly soluble in chloroform, and in fluorophilic solvents such as THF and diethyl ether.

**Table 1:** Solubility of **3a** and **3b** in fluorous solvents^a^.

[**3a**]\Solvent	FC-72^b^	FC-75^b^	FC-77	HFE-7100	HFE-7500	F-626

1 mM	–	–	–	+	+	+
2 mM	–	–	–	+	+	+
5 mM	–	–	–	+	+	+
10 mM	–	–	–	+	+	+
						
[**3b**]\Solvent	FC-72	FC-75	FC-77	HFE-7100	HFE-7500	F-626

1 mM	+	+	+	+	+	+
2 mM	–	–	–	+	+	+
5 mM	–	–	–	+	+	+
10 mM	–	–	–	+	+	+

^a^**3a** and **3b** were heated in solvent until a clear solution formed. This was allowed to cool to room temperature and stand. **3a** and **3b** were determined to be soluble at the recorded concentration if no precipitate was observed after 24 h. ^b^**3a** recrystallized upon cooling overnight.

Similarly, **3b** was soluble in fluorous solvents, FC-72 (perfluorohexanes), FC-75 (perfluoro-(2-perfluorobutyl)tetrahydrofuran), FC-77 (perfluorooctanes), HFE-7100 (methyl nonafluorobutyl ether), HFE-7500 (3-ethoxy-1,1,1,2,3,4,4,5,5,6,6,6-dodecafluoro-2-trifluoromethylhexane), and F-626 (1*H*,1*H*,2*H*,2*H*-perfluorooctyl 1,3-dimethylbutyl ether) at a 1mM or greater concentration [28–29]. Compound, **3b** also showed solubility in CO_2_ at a 2 wt % concentration, 3500 psi, and room temperature due to the presence of fluorous tails [26].

**Scheme 2:**
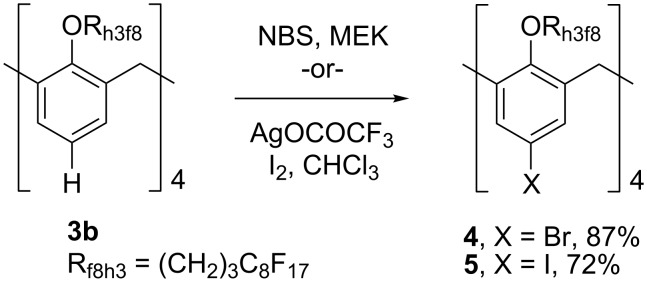
Preparation of **4** and **5**.

To expand the versatility of this scaffold, rim functionalization was explored. Halogenated calix[4]arenes have been shown to participate in a variety of organometallic processes, particularly palladium catalyzed cross coupling reactions, including Kumada, Negishi, and Suzuki processes which can be used to append aromatic rings onto the molecule [30–31]. Therefore, **3b** was treated with *N*-bromosuccinimide (NBS) in methyl ethyl ketone (MEK) [32] to give the bromide **4** in 87% yield. Correspondingly, **3b** was treated with silver trifluoroacetate [32] in the presence of iodine providing iodide **5** in 72% yield on a 1 mmol scale (Scheme 2). Results for the iodination were scale dependent; near quantitative yields could be obtained on 0.1 mmol scale preparations, while 1 mmol scale preparations showed diminished yields due to product occlusion with the precipitation of silver iodide.

**Scheme 3:**
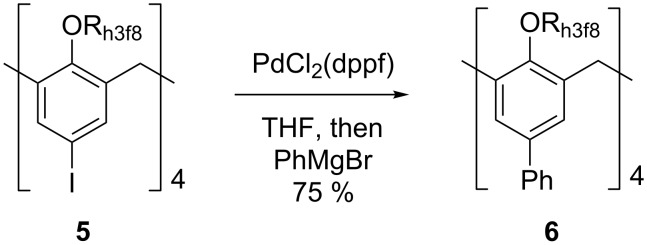
Preparation of **6**.

The reactivity of **5** in the Kumada cross-coupling reaction was next investigated. Treatment of **5** with PdCl_2_(dppf) followed by phenylmagnesium bromide provided the biaryl **6** as the only observed product in 75% yield (Scheme 3).

**Scheme 4:**
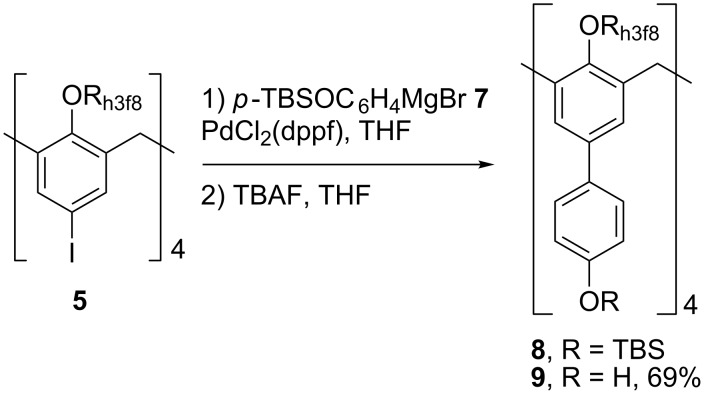
Preparation of **8** and **9**.

With simple cross coupling accomplished, coupling with a functionalized phenyl ring was investigated. Therefore, **5** was treated with an excess of Grignard **7** in the presence of PdCl_2_(dppf) to provide a mixture of two inseparable compounds, the target biaryl **8**, and the dimer of **7**, as observed by NMR spectroscopy. Without separation, the two compounds were carried on to the subsequent TBS cleavage with TBAF to provide the free tetrol **9** after column chromatography in 69% yield over two steps (Scheme 4).

**Figure 2:**
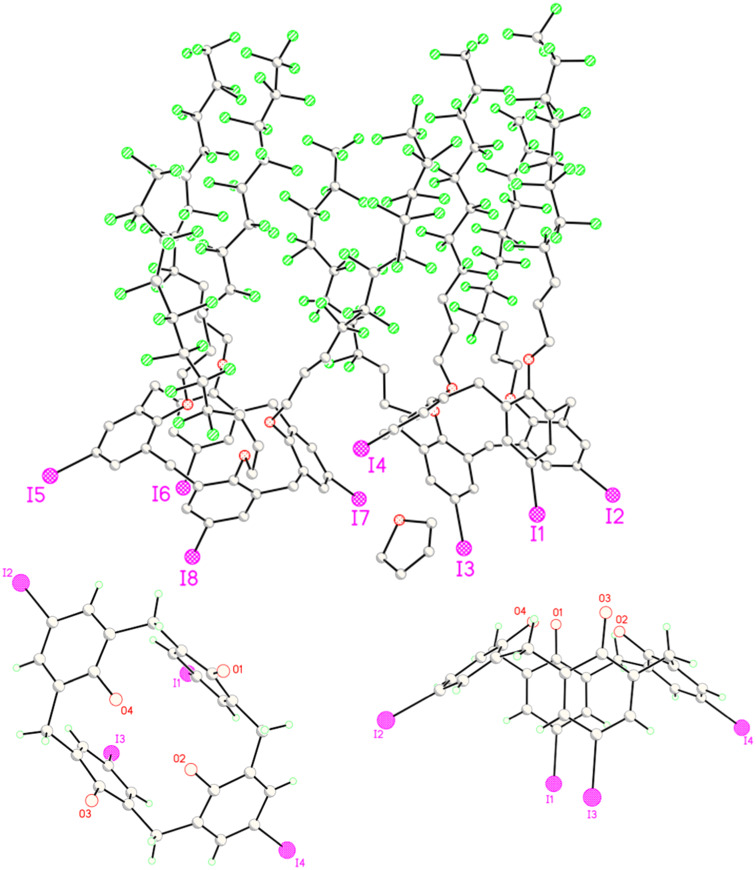
Full (top), top (bottom left)^a^, and side (bottom right)^a^ views of **5**; ^a^fluorous chains omitted for clarity.

The conformations of these new fluorous calixarenes are important to understand for projected applications. The cone conformation of **3b** was supported by peak symmetry observed in similar examples [8,31] by ^1^H NMR spectroscopy. Accordingly, the derived products should also have cone conformations. Crystals of **5** were grown by slow evaporation from a solution in THF, and one of these provided the X-ray structure in Figure 2.

Two crystallographically independent calixarene molecules made up the asymmetric unit, each molecule having a similar calix[4]arene ring and differing in the number and location of the gauche bonds in the (perfluorooctyl)propyl chains. The asymmetric unit also contained one molecule of THF. Like other reported calixarenes [33], **5** exists in a pinched cone conformation with *C*_2_*_v_* cavity symmetry in the solid state. Its cavity volume is about 81 Å^3^.

Although the calixarenes **3**–**5** have an inherent cavity in this conformation, the cavity volume and surface area are small, thus limiting the scope of possible host-guest interactions. Increasing the depth of the cavity by coupling **5** with aromatic rings to give **9** allows for host-guest interactions involving larger substrates. This modification increases the versatility of the scaffold and the variety of host-guest interactions that can occur in ion binding [8] and capsule formation [34]. Likewise, introduction of hydrogen bonding groups like those of **9** are crucial for achieving interactions with various substrates [35–36].

Coupling an aromatic ring onto the upper-rim of the fluorous calixarene led to an increase in fluorescence emission (as observed qualitatively on TLC). An increase in fluorescence emission was observed with **7**, **8**, and **9** as compared to the single aryl ring analogs, and allows for better applications of the scaffold as a sensor [8,37].

## Conclusion

Deep-cavity functionalized fluorous calix[4]arenes that are locked in the cone conformation have been synthesized. These molecules are soluble in several fluorous solvents, and show promise as fluorescent sensors. Introducing the hydroxyl functionality onto these molecules provides a scaffold with a deep cavity and hydrogen bonding functional groups for molecular recognition interactions.

## Supporting Information

File 1 Experimental Procedures, Characterization Data and Copies of Spectra

File 2 Crystal structure data for **5**.
