# Supplementary material for: Synthesis of deep-cavity fluorous calix[4]arenes as molecular recognition scaffolds
Source: Beilstein J Org Chem. 2008 Oct 20;4:36. doi: 10.3762/bjoc.4.36 (PMC2587948; doi:10.3762/bjoc.4.36)
Supplement: File 1 — Experimental Procedures, Characterization Data and Copies of Spectra [file Beilstein_J_Org_Chem-04-36-s001.pdf]

–Supporting Information–

Experimental Procedures, Characterization Data

and Copies of Spectra for

Synthesis of deep-cavity fluorous calix[4]arenes as molecular recognition scaffolds

Maksim Osipov, Qianli Chu, Steven J. Geib, Dennis P. Curran*, and Stephen G. Weber*

Department of Chemistry, University of Pittsburgh, Pittsburgh, Pennsylvania 15260, USA

Email: Dennis P. Curran - curran@pitt.edu, Stephen G. Weber - sweber@pitt.edu

Table of Contents:

Experimental and spectroscopic data for compounds **3a** – **9**..S2

Copies of ^{1}H, ^{13}C and ^{19}F NMR spectra S7

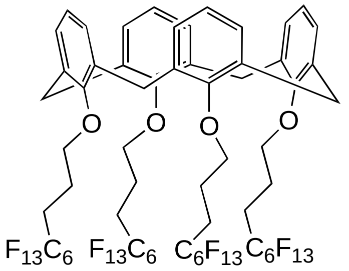

25,26,27,28-(4',4',5',5',6',6',7',7',8',8',9',9',9'-tridecafluorononyloxy)calix[4]arene (3a).
To a flask containing calix[4]arene (1.00 g, 2.36 mmol) and NaH (566 mg, 23.60 mmol) in an ice bath, was slowly added anhydrous DMF (40 mL). Hydrogen gas evolution was observed. The resulting grey suspension was stirred for 15 min. To this suspension, 3(perfluooctyl)propyl iodide (8.28 g, 16.96 mmol) was added, and the mixture was stirred for 16 h. The reaction mixture was cooled to 0 ^{o}C, and methanol (10 mL) was added slowly. After 10 min, the reaction mixture was poured onto ether (100 mL) and water (100 mL). The organic layer was washed with water (3x, 50 mL). The ether layer was dried over Na_2SO_4, and the organic layer was concentrated, and purified using column chromatography (5% EtOAc/hexane) and then recrystallized in $CHCl_3$/EtOH to provide the desired product as small transparent rhomboids (2.69 g, 61%), mp 112 °C: ^{1}H NMR (300 MHz, $CDCl_3$): δ 6.64 (m, 12H), 4.34 (d, 4H, *J* = 13.5 Hz), 3.94 (t, 8H, *J* = 6.3 Hz), 3.24 (d, 4H, *J* = 13.5 Hz), 2.31–2.06 (m, 16H); ^{13}C NMR (75.5 MHz, $CDCl_3$): δ 156.2, 135.2, 129.1, 123.3, 74.2, 31.4, 27.9 (d, *J* = 23.4 Hz), 21.51; ^{19}F NMR (282.4 MHz, $CDCl_3$): δ –81.85 (t, 3F, *J* = 9.3 Hz), –115.33 (br s, 2F), –122.91 (br s, 2F), –123.91 (br s, 2F), –124.19 (br s, 2F), –127.19 (br s, 2F); HRMS TOF ES^{+} Calc for $C_{64}H_{44}O_4$F52, 1887.231 (with Na+); Found: 1887.248.

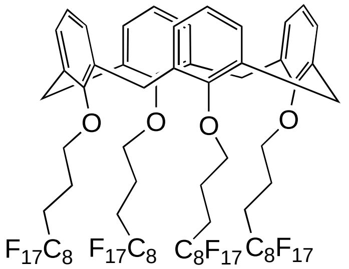

25,26,27,28-(4',4',5',5',6',6',7',7',8',8',9',9',10',10',11',11',11'-heptadecafluoroundecyloxy)-calix[4]arene (3b).

To a flask containing calix[4]arene (1.10 g, 2.60 mmol) and NaH (627 mg, 26.14 mmol) in an ice bath was slowly added anhydrous DMF (44 mL). Hydrogen gas evolution was observed. The resulting grey suspension was stirred for 15 min. To this suspension, 3(perfluooctyl)propyl iodide (11.00 g, 18.70 mmol) was added, and the mixture was stirred for 16 h. The reaction mixture was cooled to 0 °C, and methanol (10 mL) was added slowly. After 10 min, the reaction mixture was poured onto ether (100 mL) and water (100 mL). The organic layer was washed with water (3x, 50 mL). The ether layer was dried over Na_2SO_4, and the organic layer was concentrated. Slow recrystallization in $CHCl_3$/EtOH provided the desired product as a pale yellow powder (3.59 g, 61%), mp 127 °C: ^{1}H NMR (300 MHz, $CDCl_3$): δ 6.64 (m, 12H), 4.34 (d, 4H, J = 13.5 Hz), 3.94 (t, 8H, J = 6.3 Hz), 3.24 (d, 4H, J =13.5 Hz), 2.31–2.06 (m, 16H); ^{13}C NMR (75.5 MHz, $CDCl_3$): δ 156.2, 135.2, 129.1, 123.3, 74.2, 31.4, 27.9 (d, J = 23.4 Hz), 21.51; ^{19}F NMR (282.4 MHz, $CDCl_3$): δ –81.87 (t, 3F, 10.2 Hz) –115.37 (br s, 2F), –122.5 to –124.7 (m, 6F), –123.6 to –124.3 (m, 4F), –127.23 (br s, 2F).

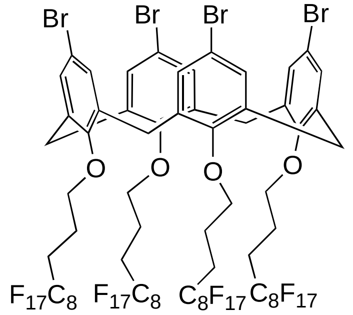

5,11,17,23-tetrabromo-25,26,27,28-tetra(4',4',5',5',6',6',7',7',8',8',9',9',10',10',11',11',11'-heptadecafluoroundecyloxy)calix[4]arene (4).

To a solution of *N*-bromosuccinimide (100 mg, 0.56 mmol), in methylethyl ketone (1.5 mL), was added fluorous calixarene **3b** (100 mg, 0.04 mmol). The mixture was stirred in the dark for 24 h. The reaction mixture was poured onto methanol (15 mL) and filtered. The residue was collected to yield the pure product as a white powder (100 mg, 87% yield), mp 171–175 °C; ^{1}H NMR (300 MHz, $CDCl_3$): δ 6.87 (s, 8H), 4.25 (d, 2H J = 13.5 Hz), 3.91 (t, 8H J = 6.3 Hz), 3.19 (d, 4H, J = 13.8 Hz), 2.26–2.03 (m, 16H); ^{13}C NMR (75.5 MHz, $CDCl_3$): δ 155.2, 136.6, 132.1, 116.9, 74.7, 31.2, 27,9 (t, J = 22.5 Hz), 21.5; ^{19}F NMR (282.4 MHz, $CDCl_3$): δ –81.78 (t, 3F, J = 9.6 Hz), –115.17 (br s, 2F), –122.2 to –123.2 (*m*, 6F), –123.72 (br s, 2F), –123.99 (br s, 4F), –127.11 (br s, 2F).

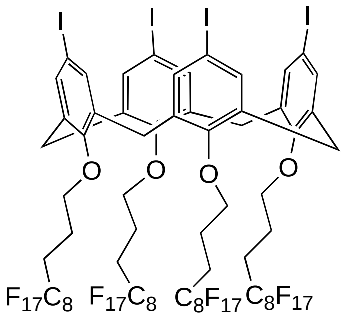

5,11,17,23-tetraiodo-25,26,27,28-tetra(4',4',5',5',6',6',7',7',8',8',9',9',10',10',11',11',11'-heptadecafluoroundecyloxy)calix[4]arene (5).

Fluorous calixarene **3b** (800 mg, 0.35 mmol) was dissolved in anhydrous $CHCl_3$ (32 mL) to this was added silver trifluoroacetate (421 mg, 1.91 mmol), and the mixture was refluxed for 4 h. Iodine (1.24 g, 4.70 mmol) was added, and the reaction mixture was stirred for 24 h. The reaction mixture was filtered, and the residue (AgI) was washed with chloroform. The filtrate was washed with saturated $Na_2S_2O_3$ (2x, 20 mL), and water (3x 20 mL). The organic layer was dried, and concentrated to a small volume (~10 mL). Methanol (100 mL) was added, and the resulting precipitate was allowed to settle for 20 min. The precipitate was filtered, and washed with methanol (3x, 30 mL) to give the desired product as a white powder (704 mg, 72% yield), mp 184.5–186 °C; ^{1}H NMR (300MHz, $CDCl_3$): δ 7.06 (s, 8H) 4.19 (d, 4H, *J* = 13.5 Hz), 3.90 (t, 8H, *J* = 6.3 Hz), 3.16 (d, 4H, *J* = 13.5 Hz), 2.35–2.03 (m, 16H); ^{13}C NMR (75.5 MHz, $CDCl_3$): δ 155.9, 138.1, 136.8, 87.7, 74.6, 30.7, 27.7 (t, *J* = 22.7 Hz); ^{19}F NMR (282.4 MHz, $CDCl_3$): δ –81.77 (t, 3F, *J* = 9.6 Hz), –115.23 (br s, 2F), –121.9 to –123.2 (m, 6F), –123.3 to –124.2 (m, 4F), –127.16 (br s, 2F).

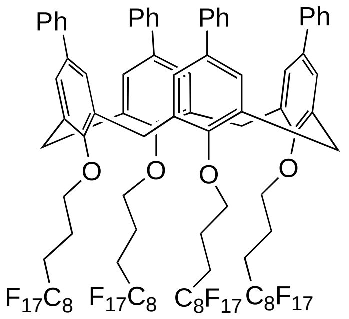

5,11,17,23-tetraphenyl-25,26,27,28-tetra(4',4',5',5',6',6',7',7',8',8',9',9',10',10',11',11',11'-heptadecafluoroundecyloxy)calix[4]arene (6).

Iodocalixarene **5** (100 mg, 0.036 mmol), and 1,1'-*bis*(diphenylphosphino)ferrocene-palladium(II)dichloride dichloromethane complex (2 mg, 2.7 μmol) were dissolved in anhydrous THF (3.2 mL). The mixture was heated to 40 °C until a clear solution formed. To this was added dropwise phenylmagnesium bromide (3M in Et_2O, 0.39 mmol, 0.129 mL). The mixture was refluxed for 16 h, and then cooled to room temperature. Methanol (1 mL), and 1M HCl (1 mL) were added to destroy unreacted Grignard. The mixture was extracted into ether (3x, 10 mL), dried, and concentrated to dryness. The resulting solid was purified by column chromatography (hexane then 5% ethyl acetate/hexane) to yield the desired product as a white solid (69 mg, 74.7% yield). mp 175–177 °C; ^{1}H NMR (300 MHz, $CDCl_3$): δ 7.12 (br s, 20H), 6.98 (s, 8H), 4.47 (d, 4H, *J* = 13.2 Hz), 4.05, (t, 8H), 3.39 (d, 4H, *J* = 13.5 Hz), 2.27 (m, 4H); ^{13}C NMR (75.5 MHz, $CDCl_3$): δ 157.4, 155.8, 136.7, 135.8, 133.4, 128.6, 127.7, 116.8, 74.9, 32.0, 28.2 (*t*, *J* = 23.3 Hz), 22.1; ^{19}F NMR (282.4 MHz, $CDCl_3$): δ –81.7 (t, 3F, *J* = 9.3 Hz), –115.1 (br s, 2F), –122.4 to –123.2 (m, 6F), –123.5 to –124.2 (m, 4F), –127.1 (br s, 2F).

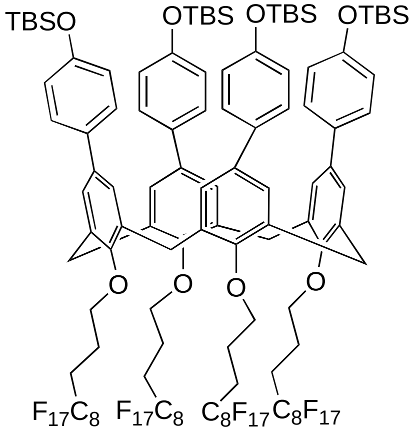

5,11,17,23-tetra-(4'-*tert*-butyldimethylsilyloxyphenyl)-25,26,27,28-tetra(4',4',5',5',6',6',7',7',8',8',9',9',10',10',11',11',11'-heptadecafluoroundecyloxy)calix[4]arene (8).

Iodocalixarene **5** (1.00 g, 0.36 mmol), and 1,1'-*bis*(diphenylphosphino)ferrocene-palladium(II)dichloride dichloromethane complex (29 mg, 0.04 mmol) were dissolved in anhydrous THF (20 mL). The mixture was heated to 40 °C until a clear solution formed. To this was added TBSO-Ph-MgBr[1] (1.03 g, 3.6 mmol) in anhydrous THF (10 mL), and the mixture was refluxed for 16 h, and then cooled to room temperature. Methanol (5 mL), and 1M HCl (5 mL) were added to destroy unreacted Grignard. The mixture was extracted into ether (3x, 30

1 Freshly prepared by refluxing (4-bromophenoxy)(*tert*-butyl)dimethylsilane in THF with Mg turnings.

mL), dried, and concentrated to dryness. The resulting solid was purified by column chromatography (hexane, then 2% ethyl acetate/hexane) to yield two inseparable compounds (target product **8** and the biaryl resulting from dimerisation of TBSO-Ph-MgBr) as a white solid (1.02 g). This mixture was carried over to the next step without further purification: ^{1}H NMR (300 MHz, $CDCl_3$): δ 7.07 (d, 8H, *J* = 8.4 Hz), 6.94 (s, 8H), 6.70 (d, 8H, *J* = 8.4 Hz), 4.45 (d, 4H, *J* = 12.9 Hz), 4.04 (t, 8H, *J* = 6.6 Hz), 3.36 (d, 4H, *J* = 13.2 Hz) 2.41–2.13 (m, 16H) 0.986 (s, 36H,) 0.194 (s, 24H).

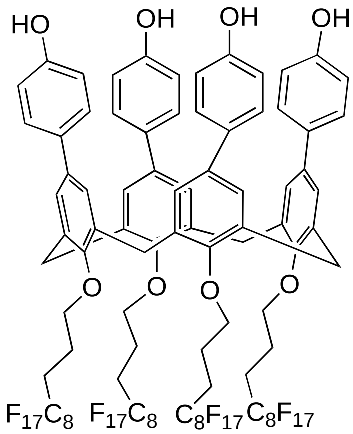

5,11,17,23-Tetra-(4'-hydroxyphenyl)-25,26,27,28-tetra(4',4',5',5',6',6',7',7',8',8',9',9',10',10',11',11',11'-heptadecafluoroundecyloxy)calix[4]arene (9).

To an ice bath cooled solution of **8** (400 mg, 0.13 mmol) in anhydrous THF (9.2 mL) was added tetrabutylammonium fluoride (1M in THF, 0.64 mL, 0.64 mmol). The pink reaction mixture was warmed to room temperature, then brought to a reflux for 4 h. The mixture was cooled to room temperature, and poured onto NH_4Cl (20 mL). The mixture was extracted into ether (3x 20 mL) and washed with water (3x 20 mL). The organic layer was dried and concentrated. The white solid was trituated with hot methanol to remove tetrabutylammonium salts, and filtered. The resulting solid was suspended in chloroform and filtered to provide the desired product as a while solid (233.5 mg, 69% yield over 2 steps), mp ~235 °C (decomp); ^{1}H NMR (300 MHz, acetone-d_6): δ 8.11 (br s, 4H) 7.07 (d, 8H, *J* = 7.5 Hz), 7.04 (s, 4H), 4.57 (d, 4H, 13.2 Hz), 4.16 (t, 8H *J* = 6 Hz), 3.43 (d, 4H, 13.2 Hz), 2.55–2.34 (m, 16H); ^{13}C NMR (75.5 MHz, acetone-d_6): δ 157.4, 155.8, 136.7, 135.8, 133.4, 128.6, 127.7, 116.3, 74.92, 32.0, 29.2 (t, *J* = 23.3 Hz), 22.1; ^{19}F NMR (282.4 MHz, acetone-d_6): δ –83.6 (t, 3F *J* = 9.9 Hz), –115.3 (br s, 2F), –122.7 to –123.6, (m, 6F), –124.2 (br s, 4F), –127.6 (br s, 2F).

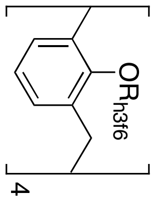
OR
h3f6
4

3a

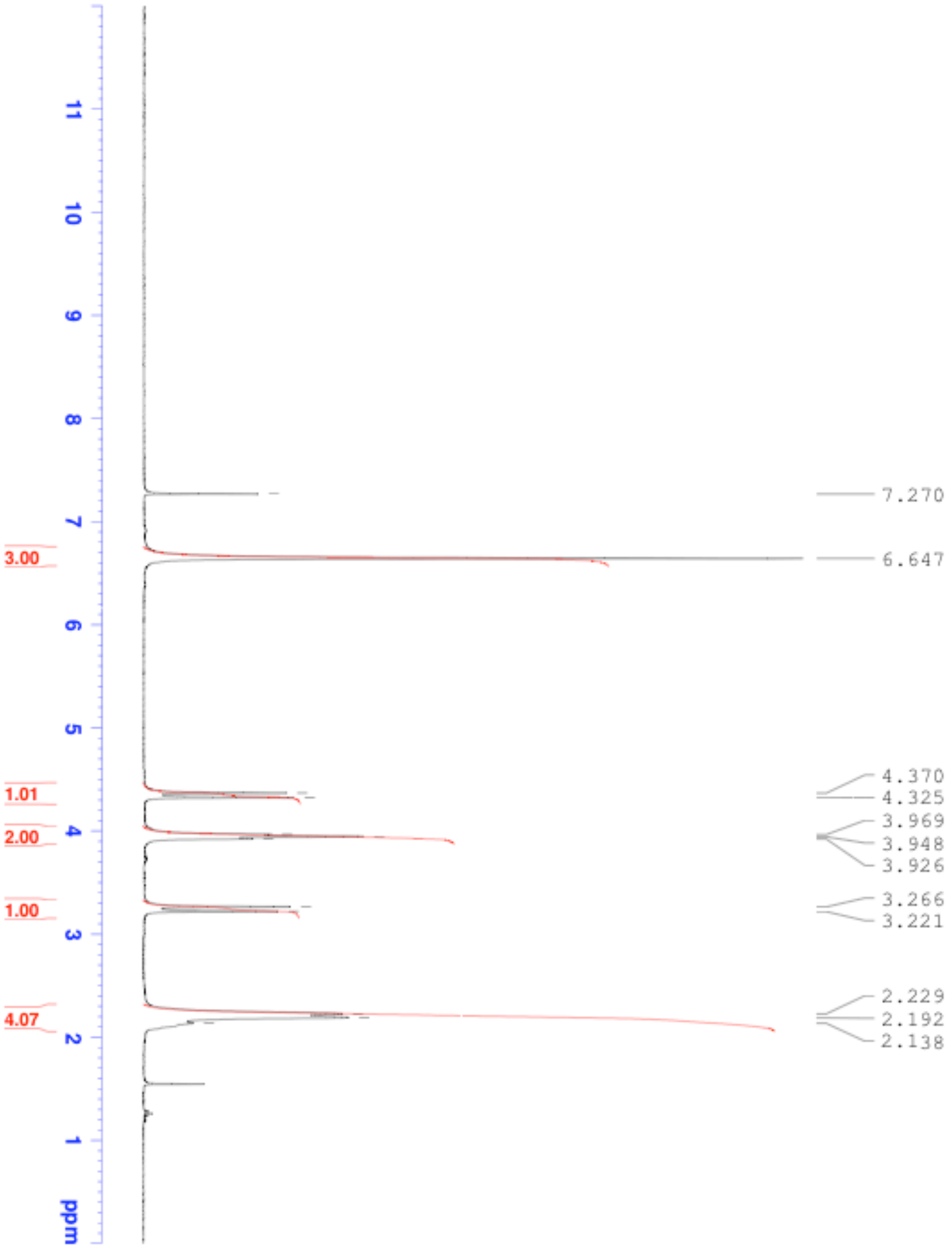
7.270
6.647
4.370
4.325
3.969
3.948
3.926
3.266
3.221
2.229
2.192
2.138
3.00
1.01
2.00
1.00
4.07
11
10
9
8
7
6
5
4
3
2
1
ppm

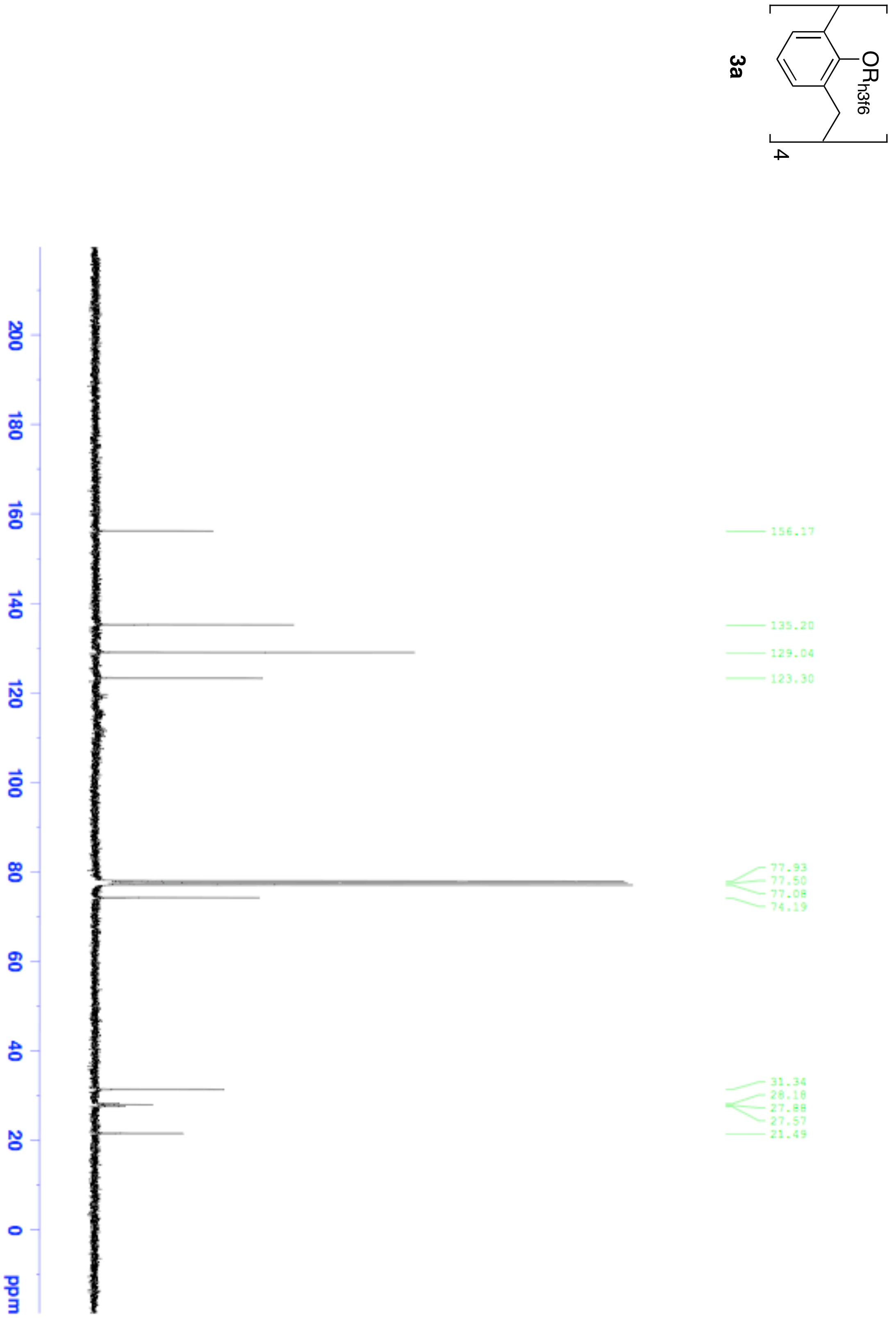
3a
ORh3f6
4
156.17
135.20
129.04
123.30
77.93
77.50
77.08
74.19
31.34
28.18
27.88
27.57
21.49
200
180
160
140
120
100
80
60
40
20
0
ppm

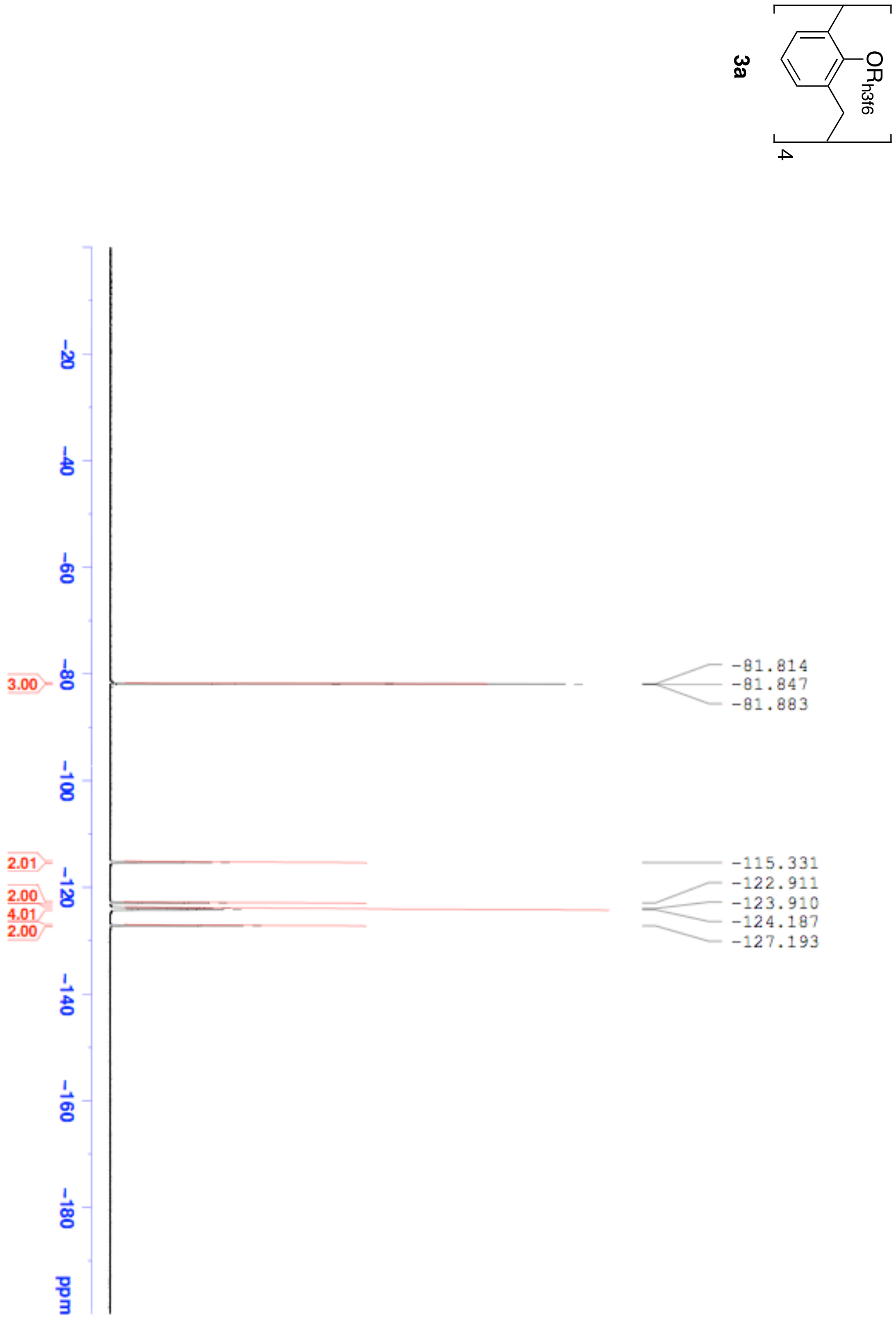
3a
OR
h3f6
4
-81.814
-81.847
-81.883
-115.331
-122.911
-123.910
-124.187
-127.193
3.00
2.01
2.00
4.01
2.00
-20
-40
-60
-80
-100
-120
-140
-160
-180
ppm

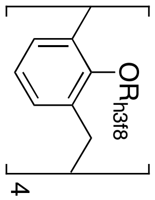
OR
h3f8
4

3b

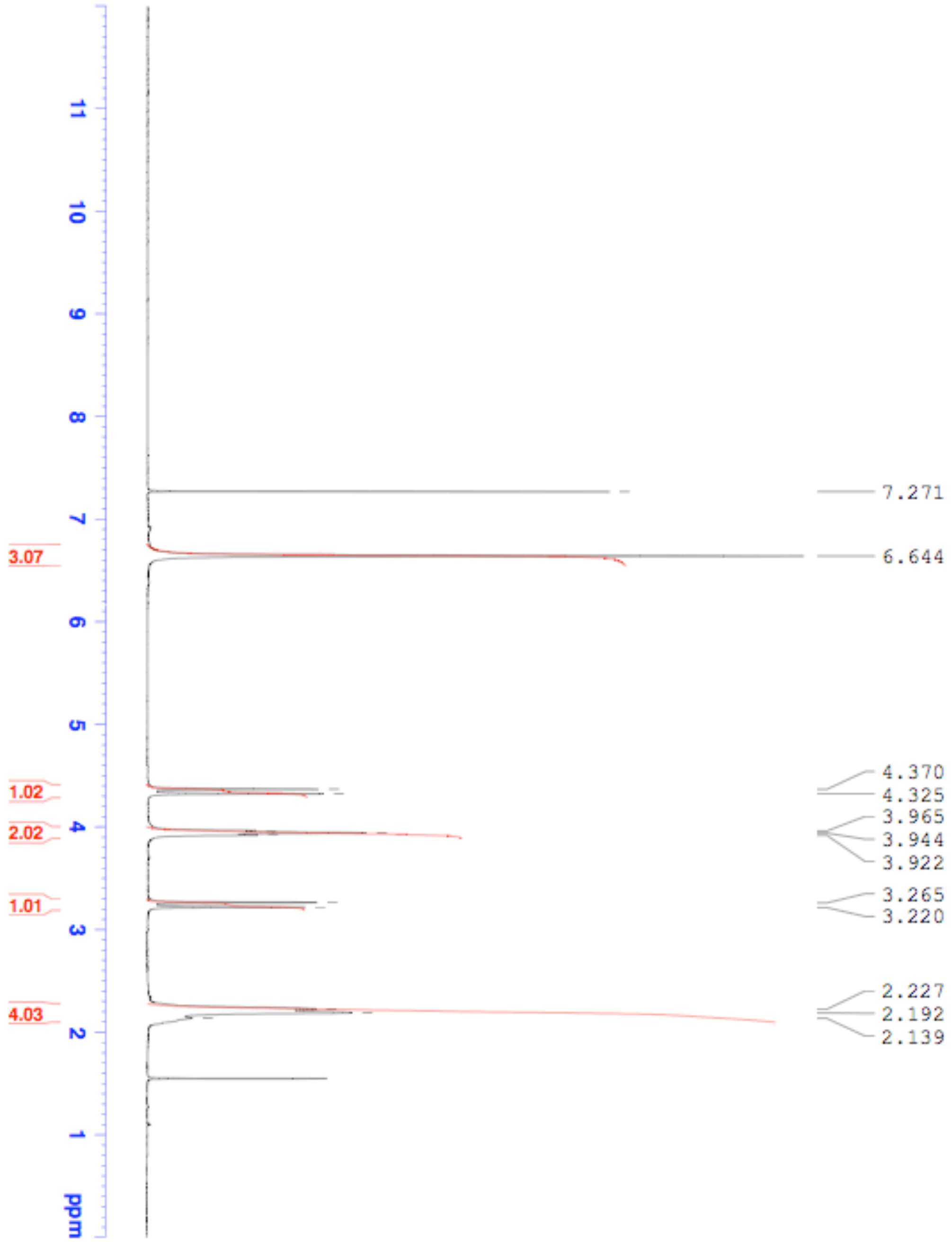
7.271
6.644
4.370
4.325
3.965
3.944
3.922
3.265
3.220
2.227
2.192
2.139
3.07
1.02
2.02
1.01
4.03
ppm

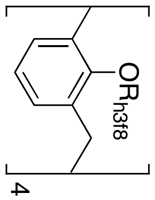
OR$_{h3f8}$
4

3b

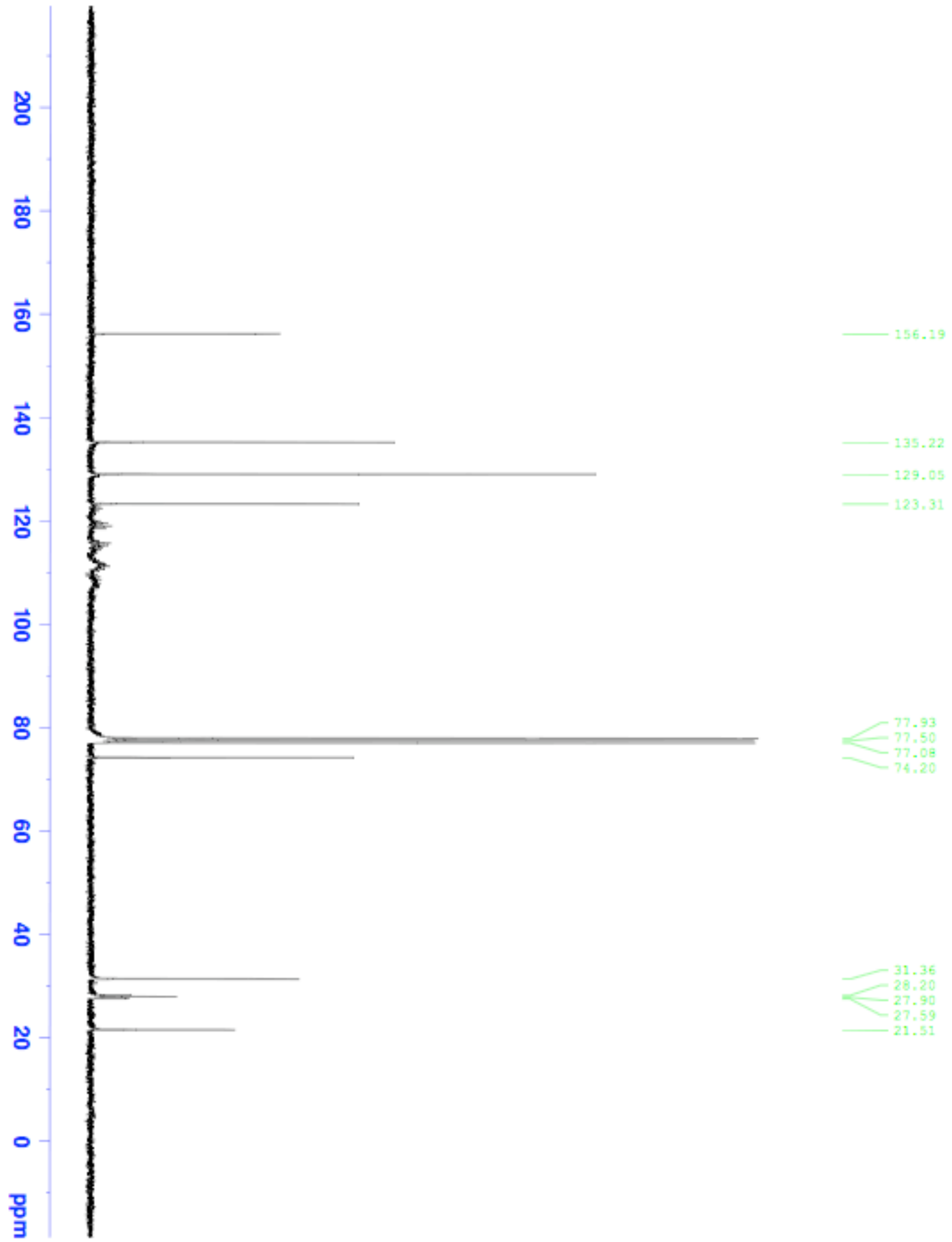
200
180
160
140
120
100
80
60
40
20
0
ppm
156.19
135.22
129.05
123.31
77.93
77.50
77.08
74.20
31.36
28.20
27.90
27.59
21.51

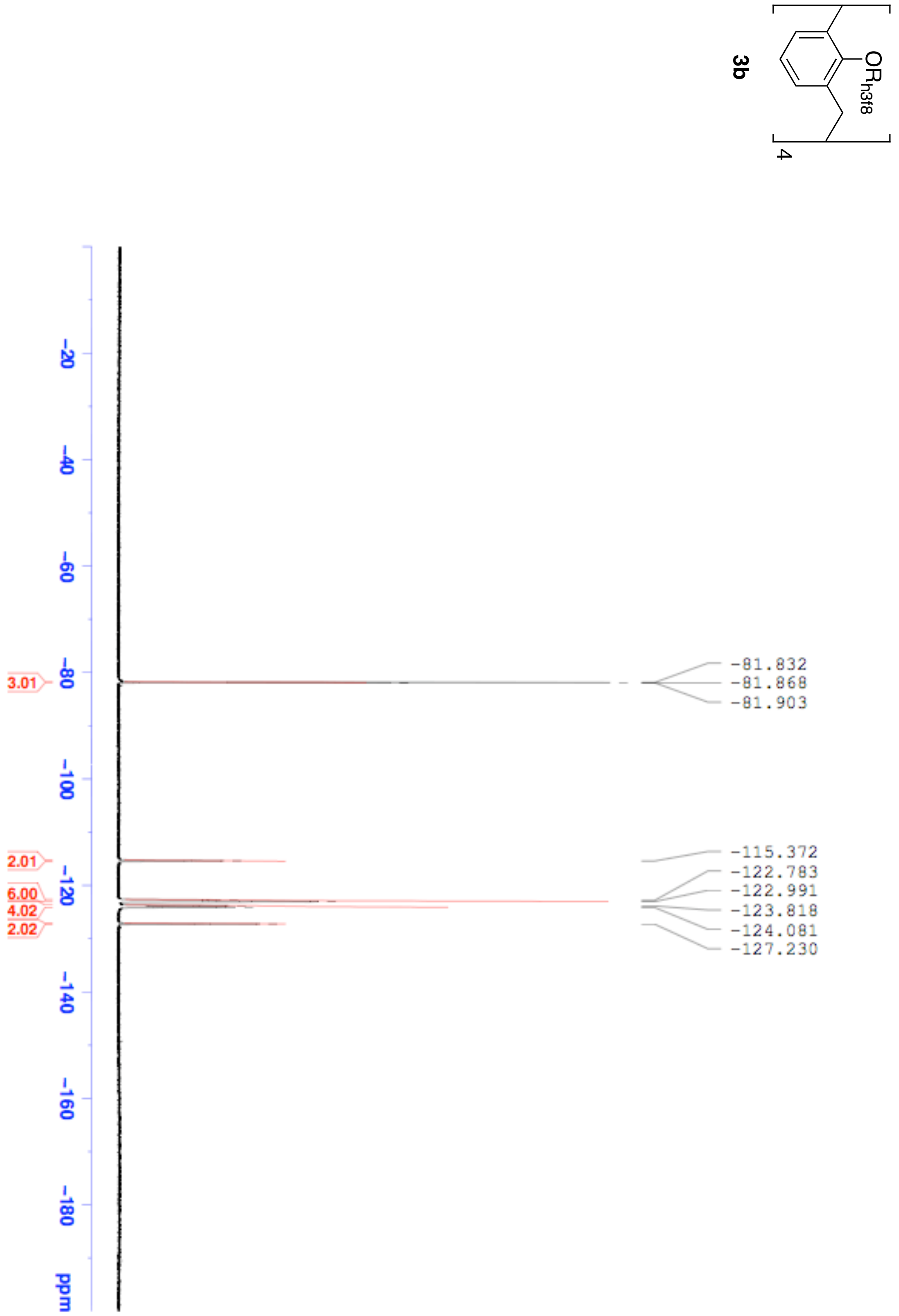
3b
ORh3f8
4
-81.832
-81.868
-81.903
-115.372
-122.783
-122.991
-123.818
-124.081
-127.230
3.01
2.01
6.00
4.02
2.02
-20
-40
-60
-80
-100
-120
-140
-160
-180
ppm

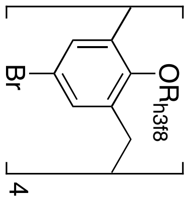

ORh3f8
Br

4

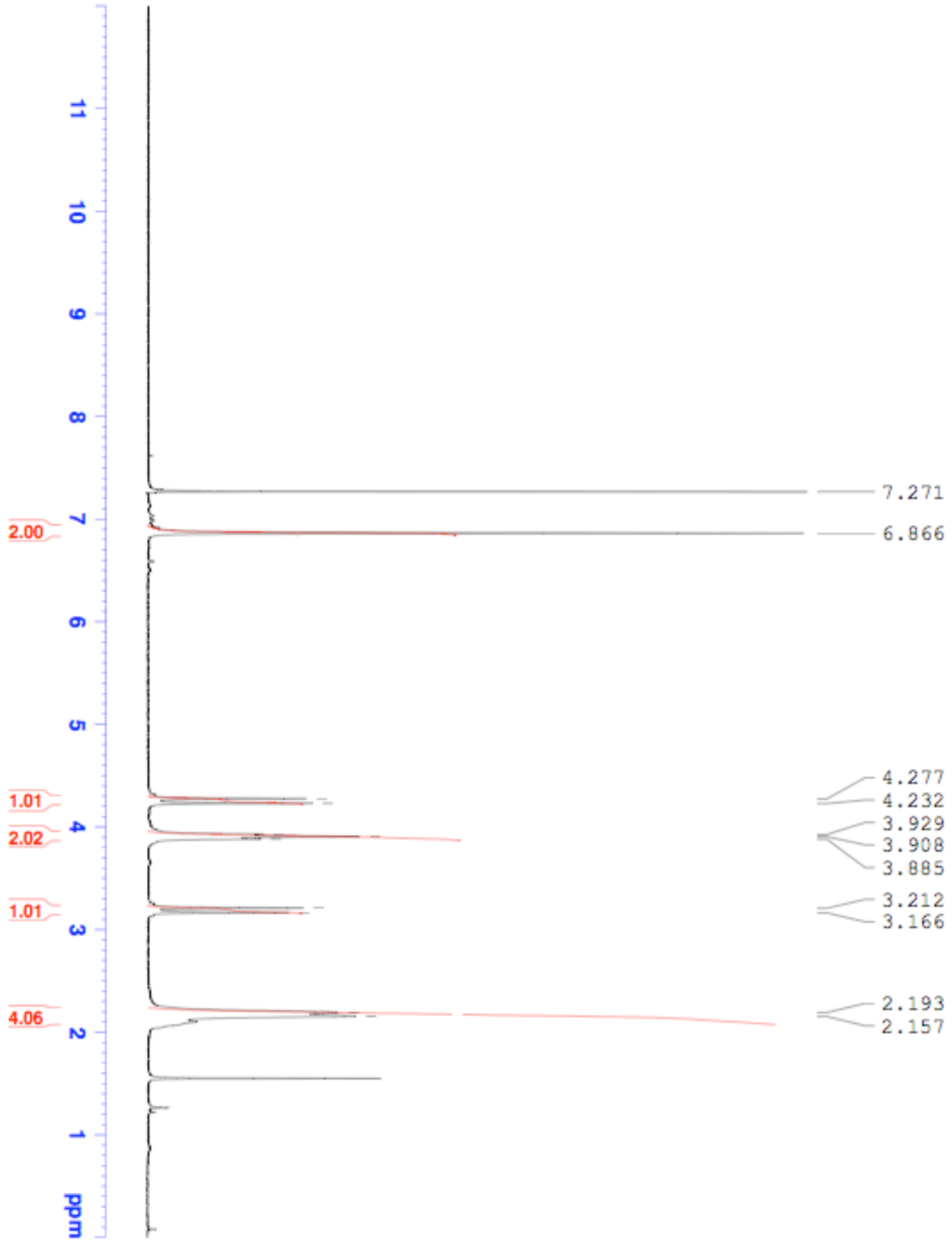

7.271
6.866
4.277
4.232
3.929
3.908
3.885
3.212
3.166
2.193
2.157
2.00
1.01
2.02
1.01
4.06
11
10
9
8
7
6
5
4
3
2
1
ppm

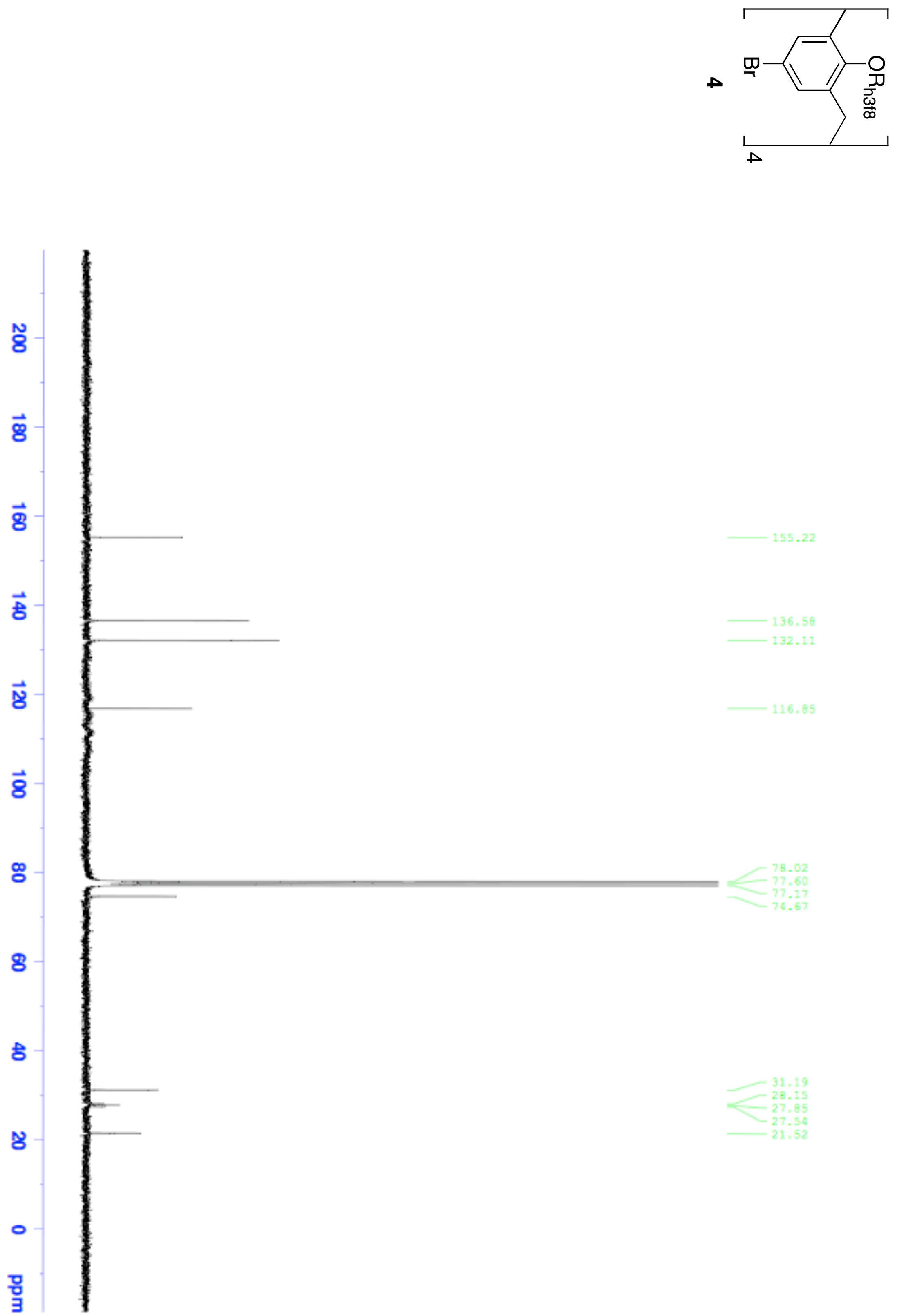
ORh3f8
Br
4
4
155.22
136.58
132.11
116.85
78.02
77.60
77.17
74.67
31.19
28.15
27.85
27.54
21.52
200
180
160
140
120
100
80
60
40
20
0
ppm

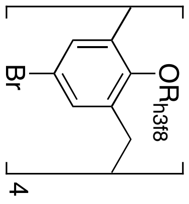
ORh3f8
Br
4

4

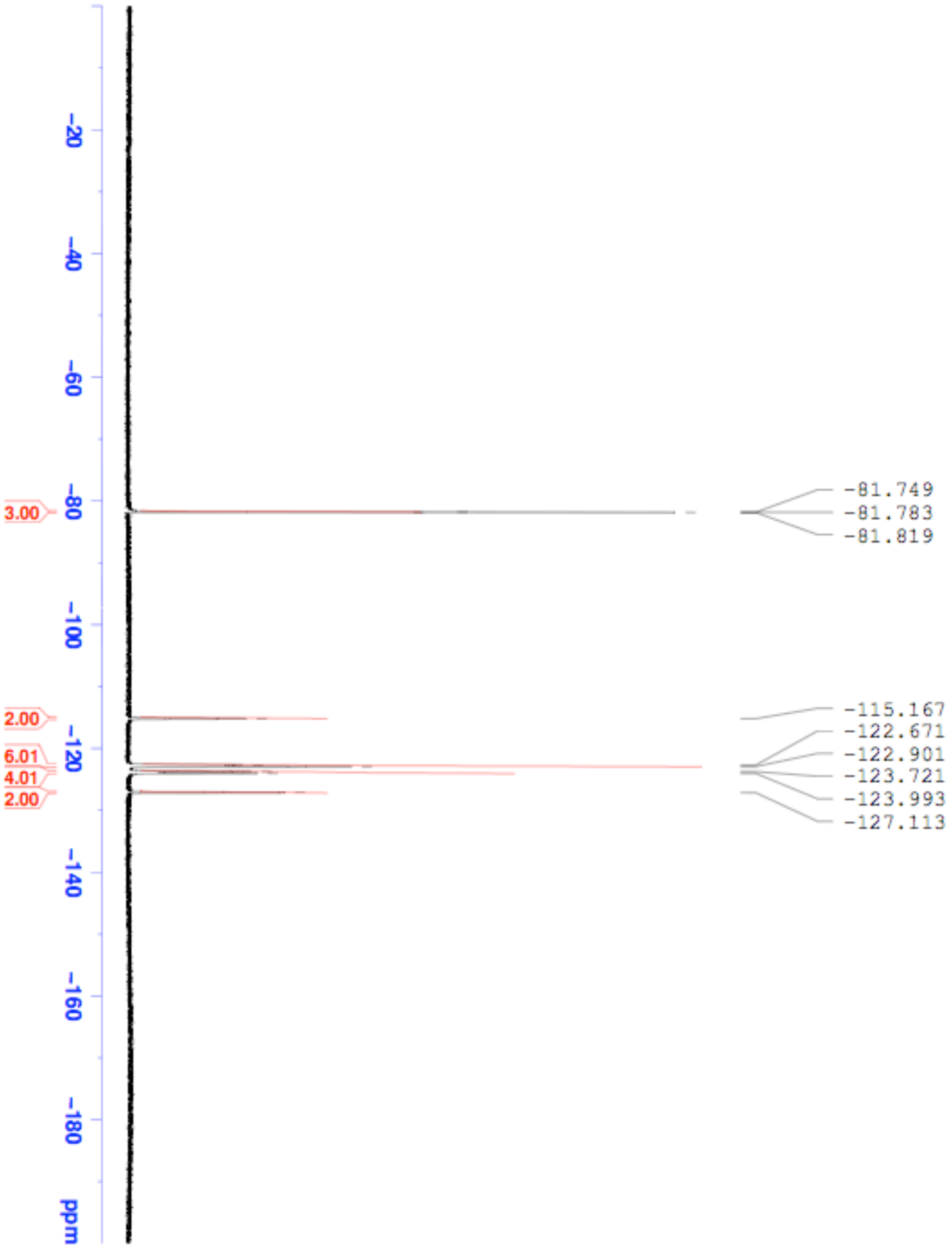
-81.749
-81.783
-81.819
-115.167
-122.671
-122.901
-123.721
-123.993
-127.113
3.00
2.00
6.01
4.01
2.00
-20
-40
-60
-80
-100
-120
-140
-160
-180
ppm

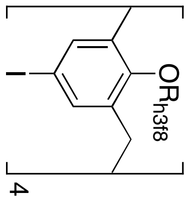

5

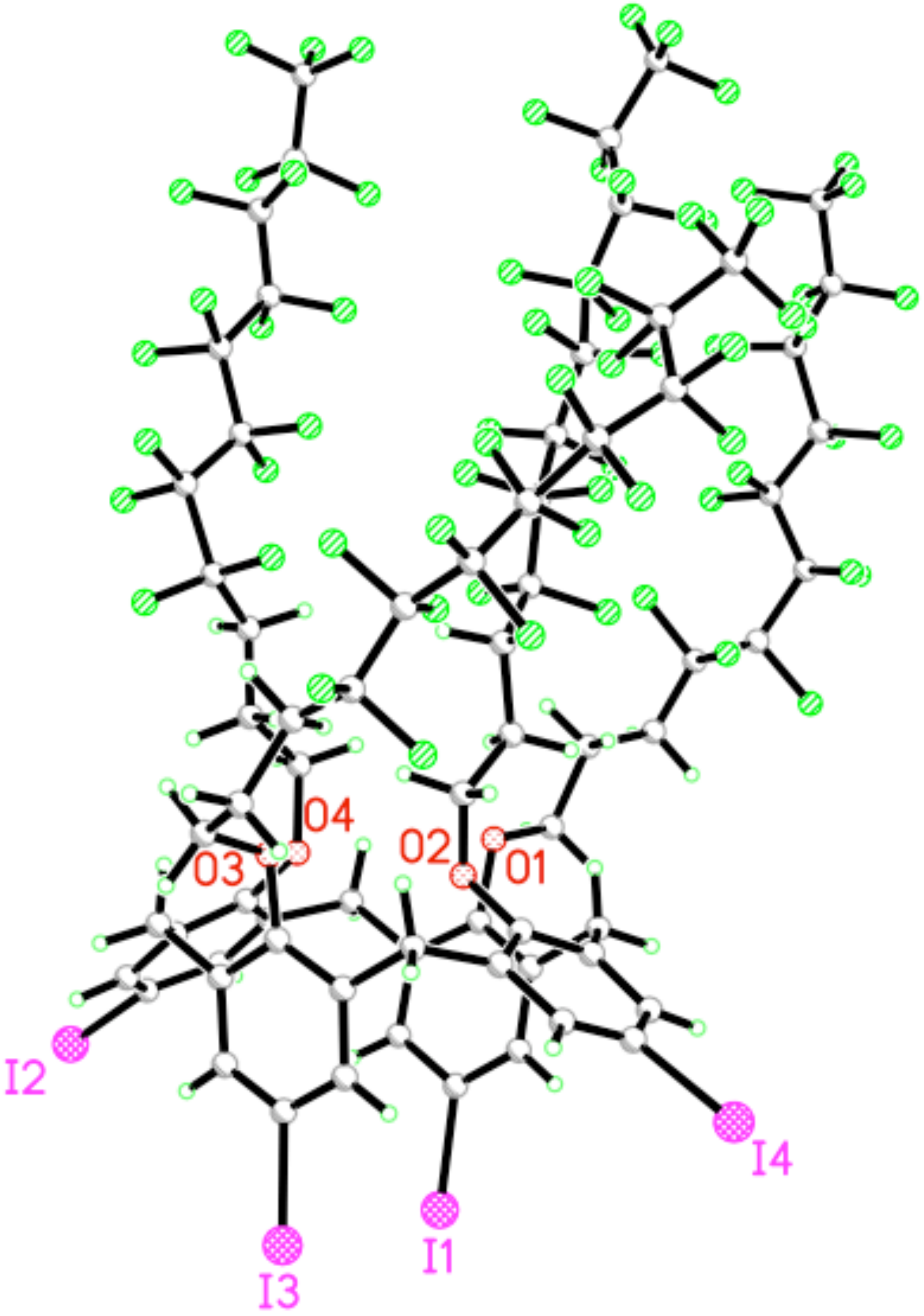

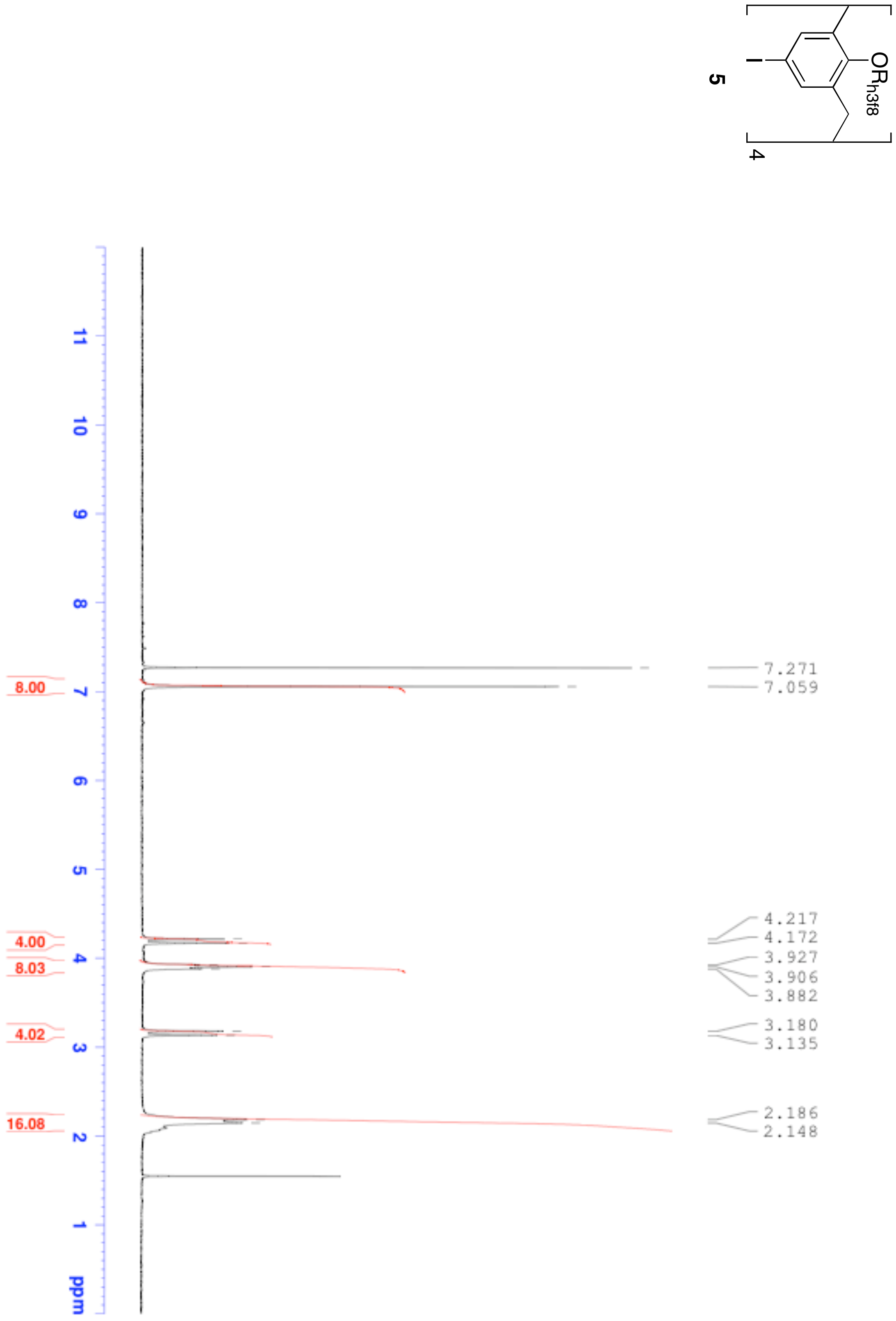

ORh3f8
I
5
4
7.271
7.059
4.217
4.172
3.927
3.906
3.882
3.180
3.135
2.186
2.148
8.00
4.00
8.03
4.02
16.08
ppm

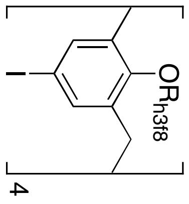
OR$_{h3f8}$
I
4

5

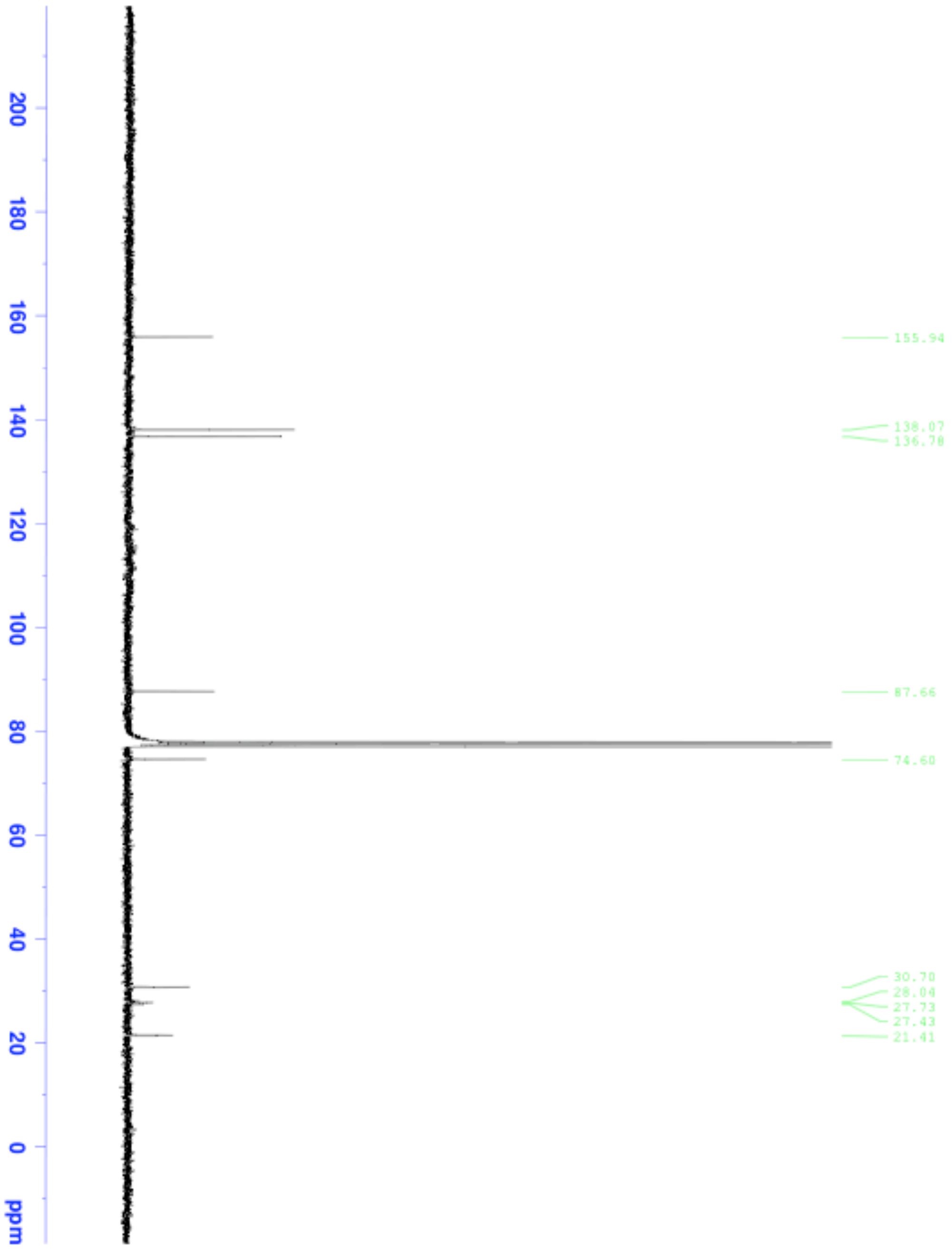
155.94
138.07
136.70
87.66
74.60
30.70
28.04
27.73
27.43
21.41
200
180
160
140
120
100
80
60
40
20
0
ppm

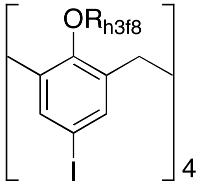
OR
h3f8
I
4

5

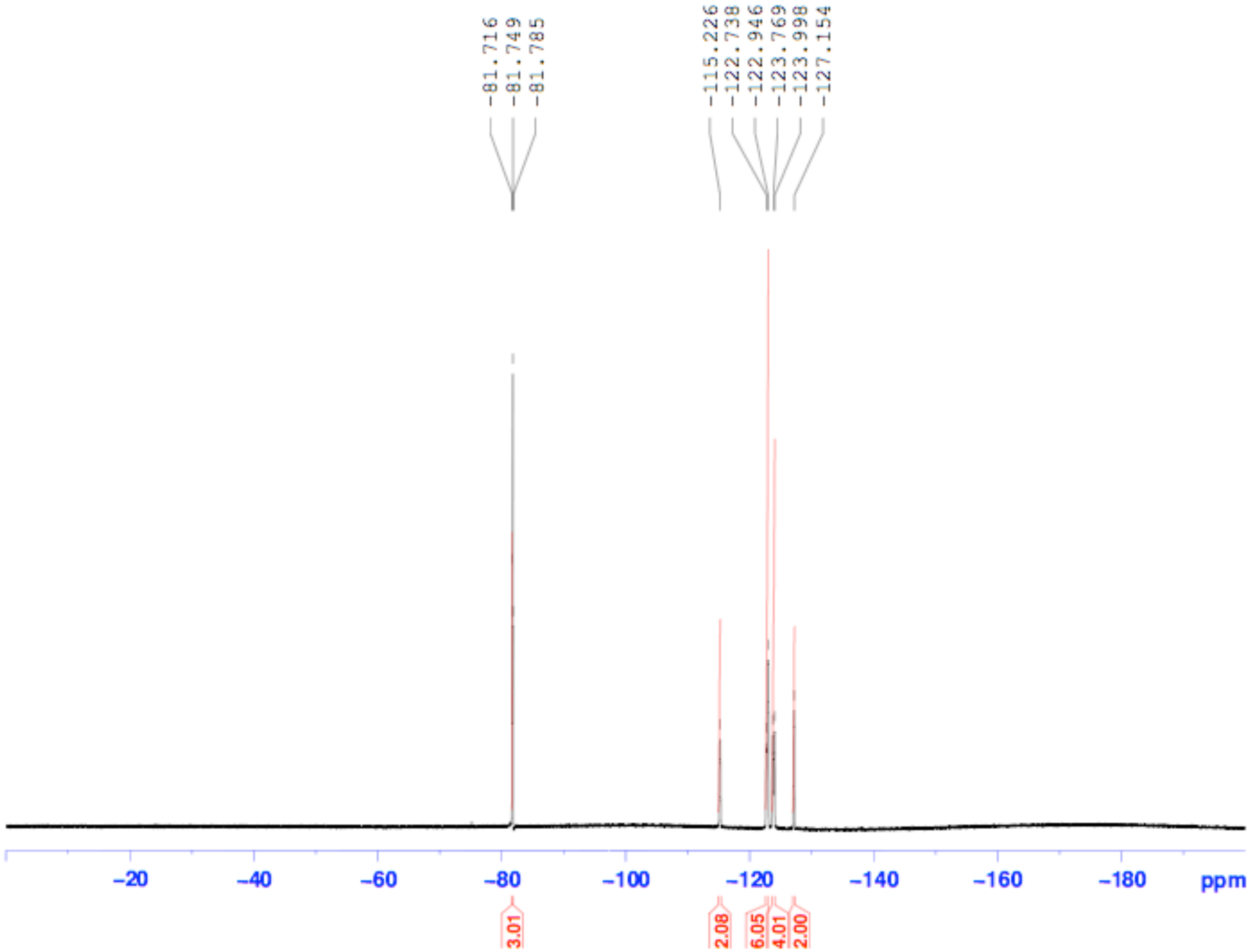
-81.716
-81.749
-81.785
-115.226
-122.738
-122.946
-123.769
-123.998
-127.154
-20
-40
-60
-80
-100
-120
-140
-160
-180
ppm
3.01
2.08
6.05
4.01
2.00

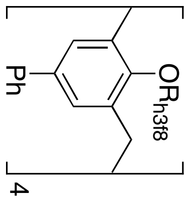
OR_h3f8
Ph
4

6

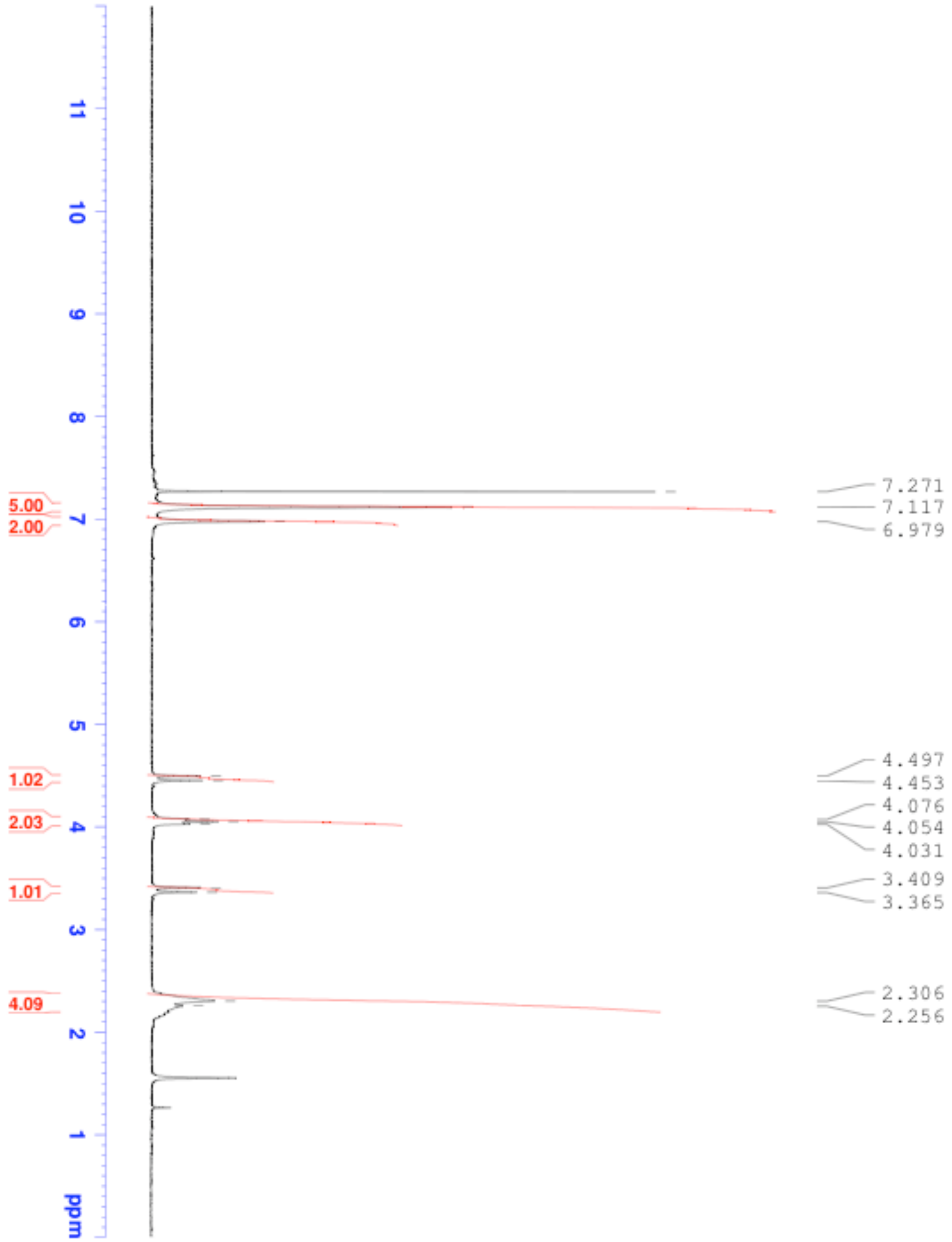
7.271
7.117
6.979
4.497
4.453
4.076
4.054
4.031
3.409
3.365
2.306
2.256
5.00
2.00
1.02
2.03
1.01
4.09
ppm

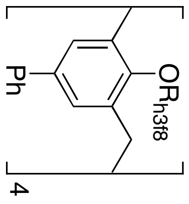
ORh3f8
Ph
4

6

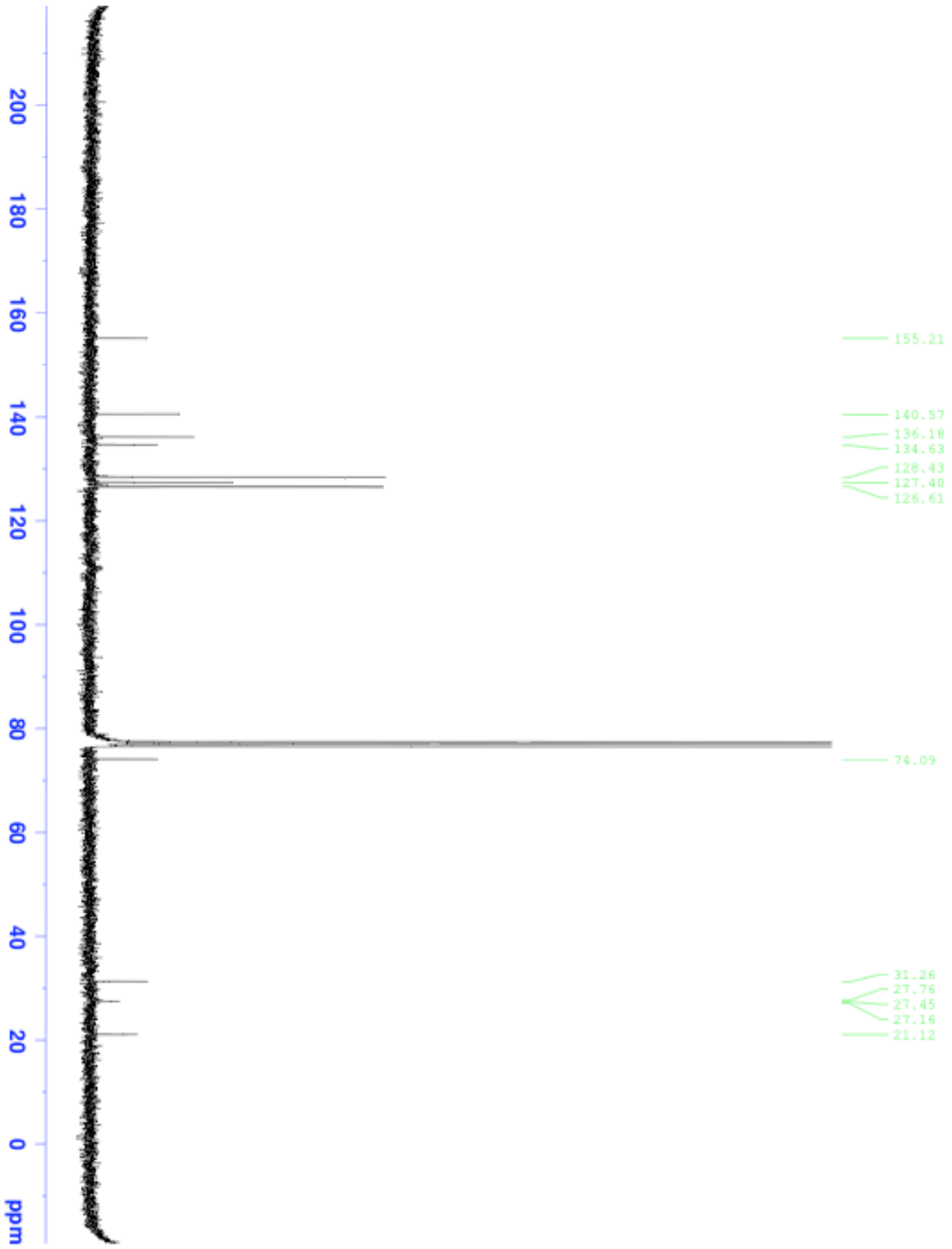
200
180
160
140
120
100
80
60
40
20
0
ppm
155.21
140.57
136.18
134.63
128.43
127.40
126.61
74.09
31.26
27.76
27.45
27.16
21.12

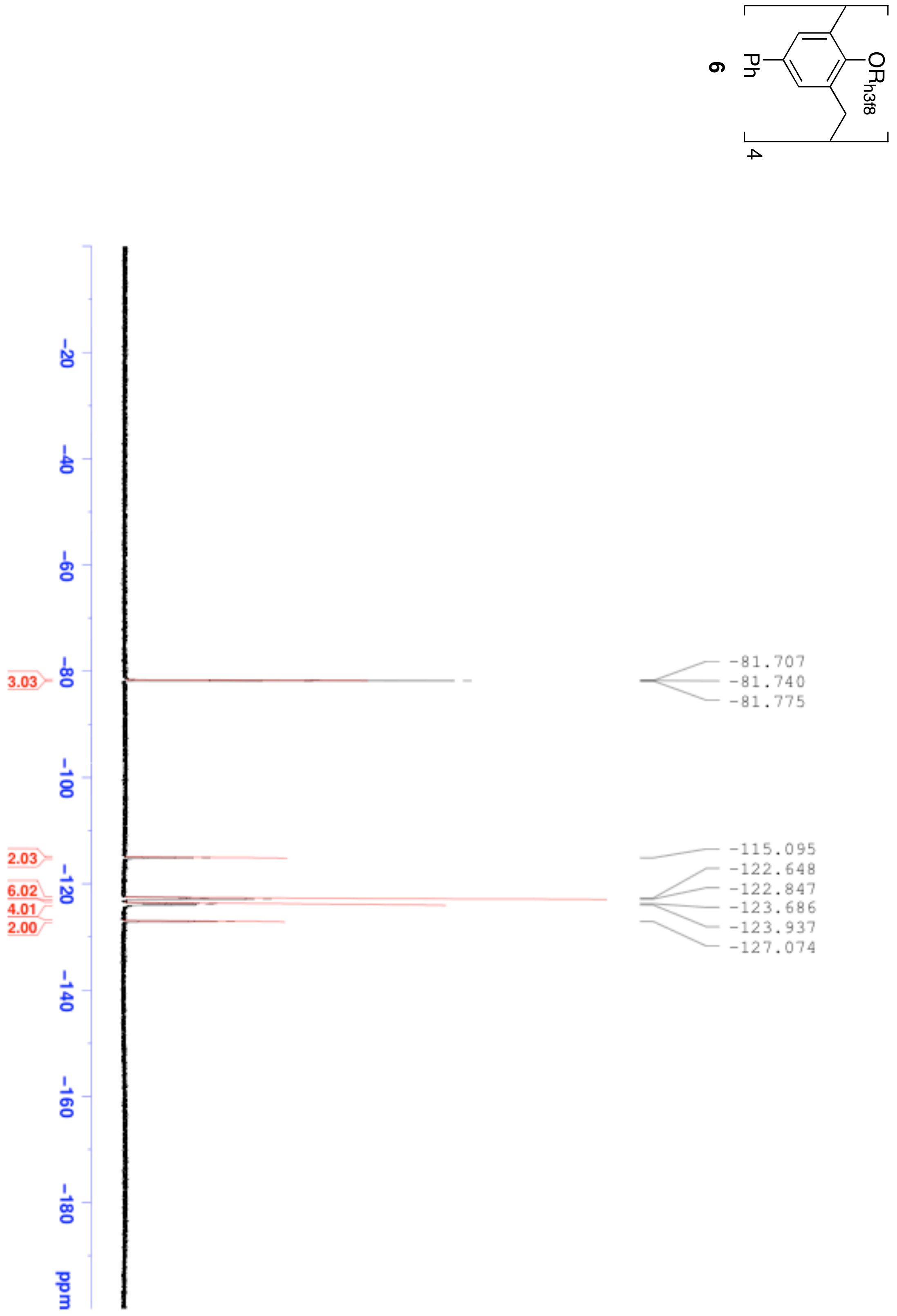
ORh3f8
Ph
6
4
-81.707
-81.740
-81.775
-115.095
-122.648
-122.847
-123.686
-123.937
-127.074
3.03
2.03
6.02
4.01
2.00
-20
-40
-60
-80
-100
-120
-140
-160
-180
ppm

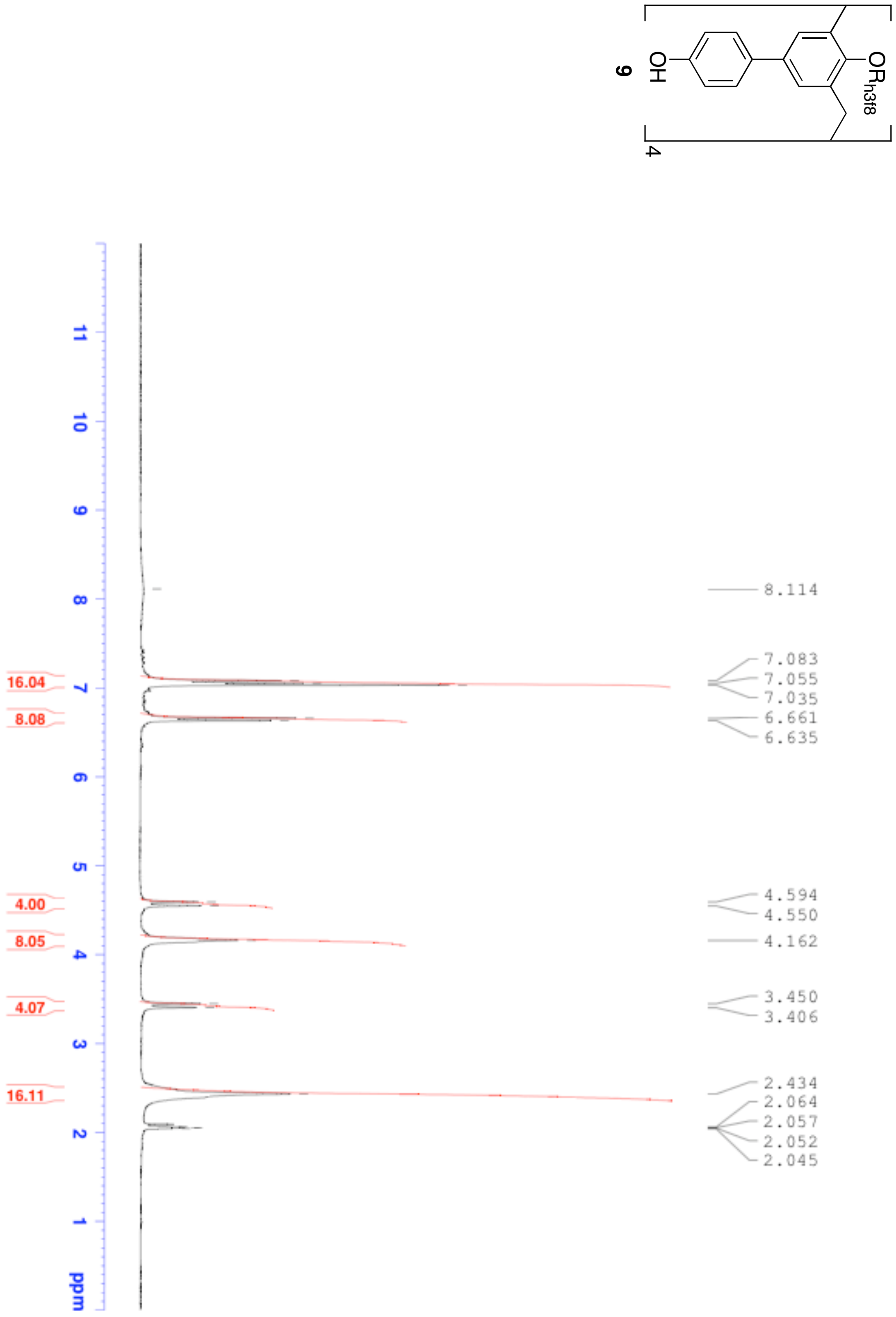

OR
h3f8
OH
4
9
8.114
7.083
7.055
7.035
6.661
6.635
4.594
4.550
4.162
3.450
3.406
2.434
2.064
2.057
2.052
2.045
16.04
8.08
4.00
8.05
4.07
16.11
11
10
9
8
7
6
5
4
3
2
1
ppm

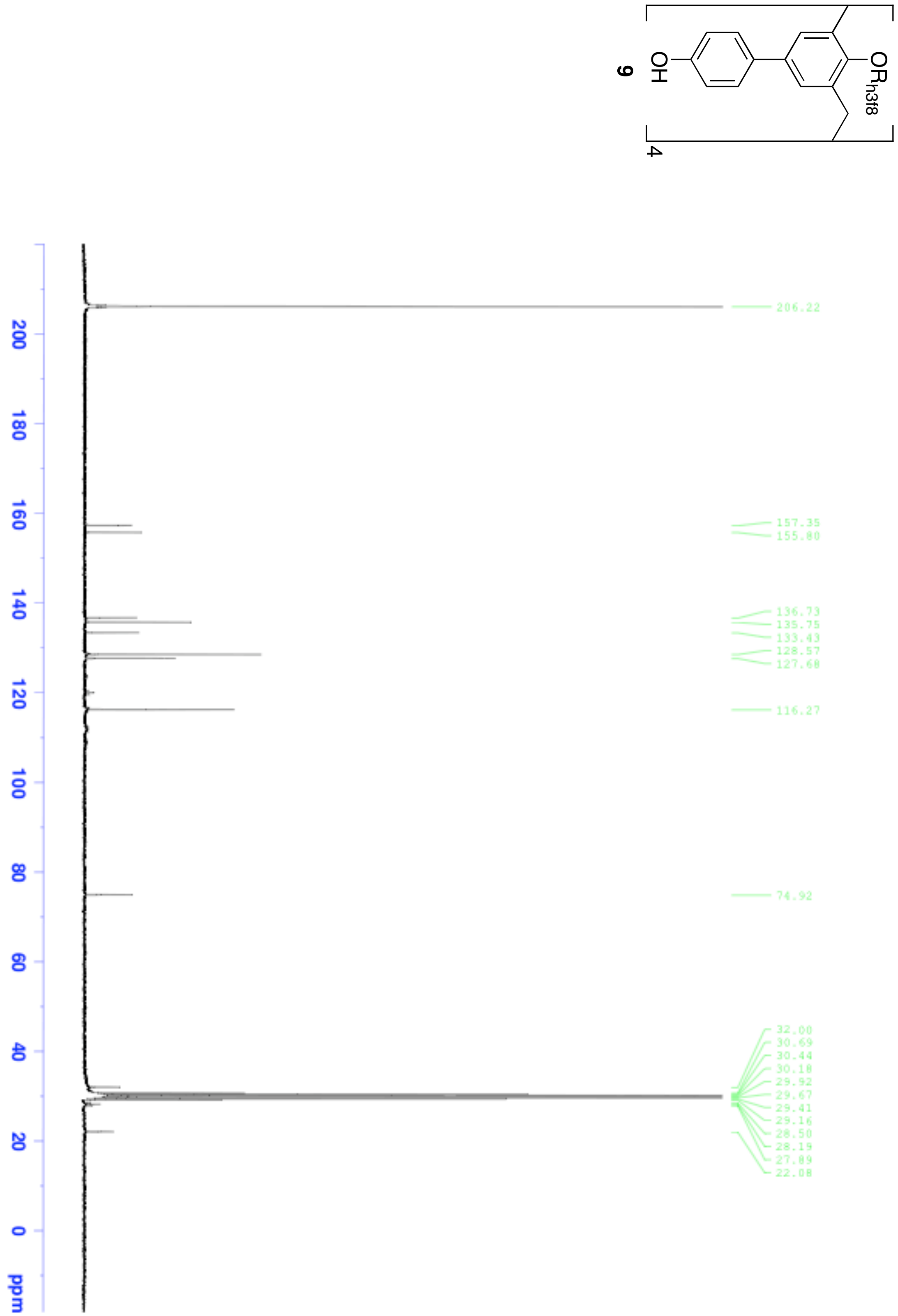
9
OH
OR h3f8
4
200
180
160
140
120
100
80
60
40
20
0
ppm
206.22
157.35
155.80
136.73
135.75
133.43
128.57
127.68
116.27
74.92
32.00
30.69
30.44
30.18
29.92
29.67
29.41
29.16
28.50
28.19
27.89
22.08

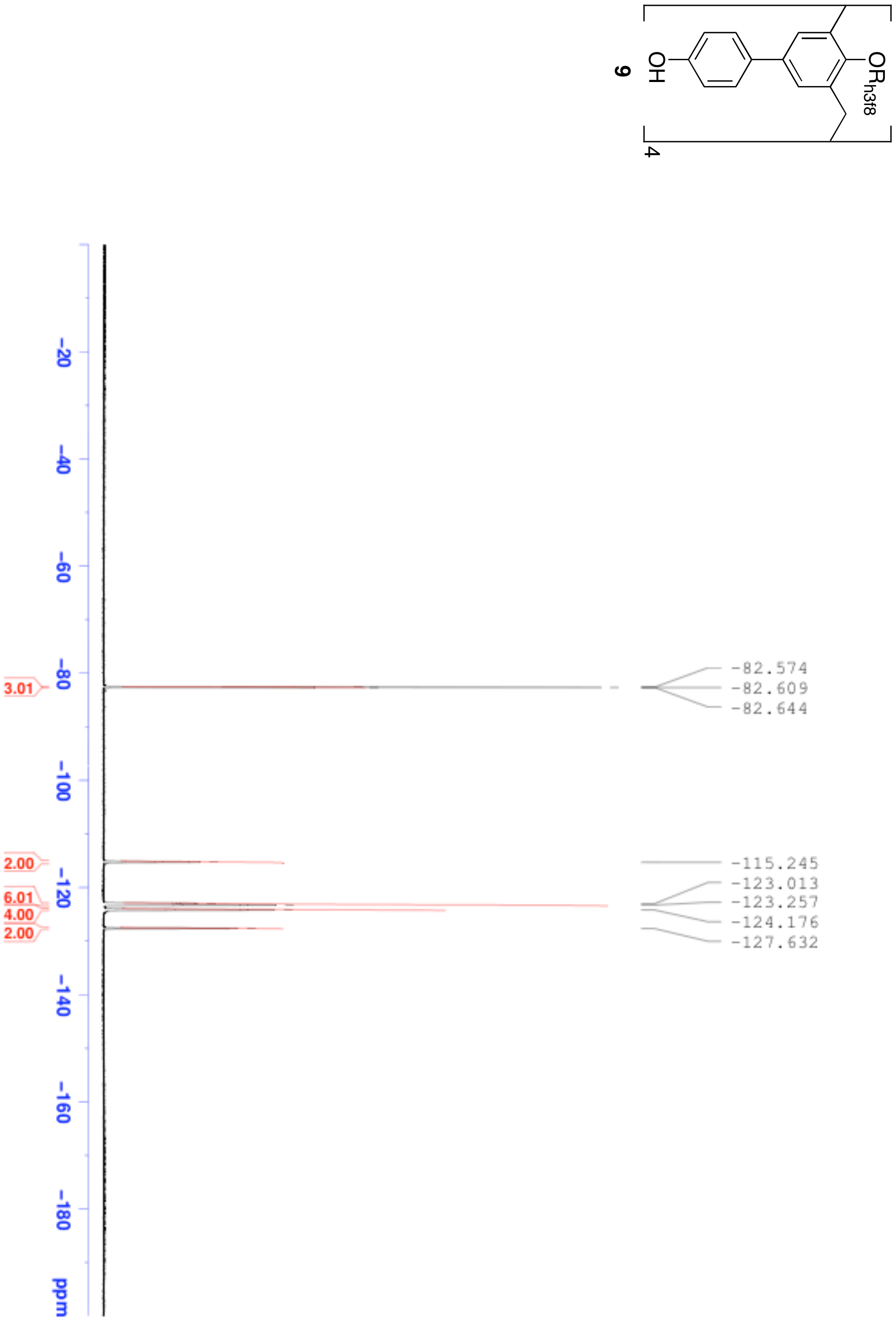

OH
OR
h3f8
4
9
-82.574
-82.609
-82.644
-115.245
-123.013
-123.257
-124.176
-127.632
3.01
2.00
6.01
4.00
2.00
-20
-40
-60
-80
-100
-120
-140
-160
-180
ppm
